# Q&A: What are exosomes, exactly?

**DOI:** 10.1186/s12915-016-0268-z

**Published:** 2016-06-13

**Authors:** James R. Edgar

**Affiliations:** Cambridge Institute for Medical Research, University of Cambridge, Hills Road, Cambridge, CB2 0XY UK

## Abstract

Exosomes are extracellular vesicles first described as such 30 years ago and since implicated in cell–cell communication and the transmission of disease states, and explored as a means of drug discovery. Yet fundamental questions about their biology remain unanswered. Here I explore what exosomes are, highlight the difficulties in studying them and explain the current definition and some of the outstanding issues in exosome biology.

## What is the current definition of an exosome?

**Fig. 1:**
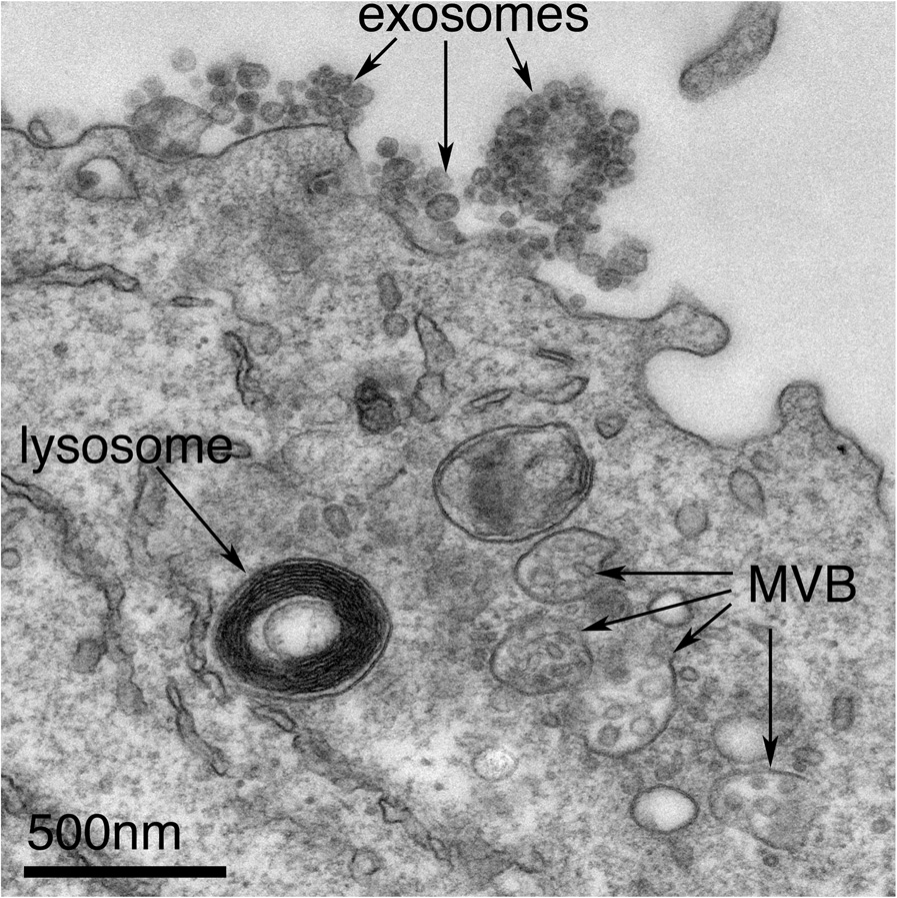
Exosomes correspond to intraluminal vesicles of multivesicular bodies. A transmission electron micrograph of an Epstein–Barr virus-transformed B cell displaying newly expelled exosomes at the plasma membrane. Multivesicular bodies (*MVB*) can be seen which can deliver content to lysosomes for degradation or can fuse with the cell surface to release intraluminal vesicles as exosomes, indicated by the *arrows* at the top of the picture

That is a very good question. Since the original description of exosomes over 30 years ago, the term has been loosely used for various forms of extracellular vesicle, muddying the field and contributing to the scepticism with which the research has sometimes been met. Exosomes are best defined as extracellular vesicles that are released from cells upon fusion of an intermediate endocytic compartment, the multivesicular body (MVB), with the plasma membrane. This liberates intraluminal vesicles (ILVs) into the extracellular milieu and the vesicles thereby released are what we know as exosomes (Fig. 1).

There are other types of microvesicle, including apoptotic bodies and ectosomes, which are derived from cells undergoing apoptosis and plasma membrane shedding, respectively. Although apoptotic bodies, ectosomes and exosomes are all roughly the same size (typically 40–100 nm) and all also contain ‘gulps’ of cytosol, they are different species of vesicles and understanding differences between them is of paramount importance but has too often been overlooked.

## How were exosomes first recognized as distinct entities?

The presence of membranous vesicles outside cells was first recognized 50 years ago, although these were originally assumed to be waste products released via shedding of the plasma membrane. The recognition of what we now call exosomes didn’t come until 1983, from studies on the loss of transferrin during the maturation of reticulocytes into erythrocytes [1]. These studies showed, by following transferrin-gold conjugates through the endocytic system, that ILVs generated in MVBs can be released to the extracellular space through fusion with the plasma membrane [2], although it was not until 1987 that the term ‘exosome’ was coined for them [3].

Even then, however, these extracellular vesicles were largely ignored, forgotten or, again, dismissed as a means of cellular waste disposal. It is only in the past decade that interest in exosomes has exploded, with a nearly tenfold increase in publications in as many years (115 in 2006, 1010 in 2015).

## Why this explosion of interest?

For at least three reasons. First, they are thought to provide a means of intercellular communication and of transmission of macromolecules between cells. Second, in the past decade, exosomes have been attributed roles in the spread of proteins, lipids, mRNA, miRNA and DNA and as contributing factors in the development of several diseases. And third, they have been proposed to be useful vectors for drugs because they are composed of cell membranes, rather than synthetic polymers, and as such are better tolerated by the host. In fact, some of the earliest exosome research indicated that they can carry the MHC–peptide complexes that are recognized by T lymphocytes [4] and that secretion of such exosomes could promote antitumour immune responses in mice in vivo [5]. Exosome therapies are now being explored in anti-cancer clinical trials and recent reports claim taxol-filled exosomes can be used to treat cancers in mice at 50-fold lower doses than conventional treatments, with the additional benefit that exosomes do not invoke an immune response [6].

Yet despite 20 years of research, the very basics of exosome biology are in their infancy and we know little of the part they play in normal cellular physiology.

## So do we know how they are generated?

Yes and no. We do know that they are made as ILVs; but first of all, not all ILVs finish up as exosomes, and second, the mechanism of their generation in endosomes is not fully understood. Most conventional membrane budding processes deform membrane from an organelle into the cytoplasm but in ILV formation the membrane buds away from the cytoplasm and into the endosome. This unconventional budding process is not limited to ILV generation but also takes place during enveloped virus budding from the cytosol and during cytokinesis [7], and it requires specialised machinery.

**Fig. 2:**
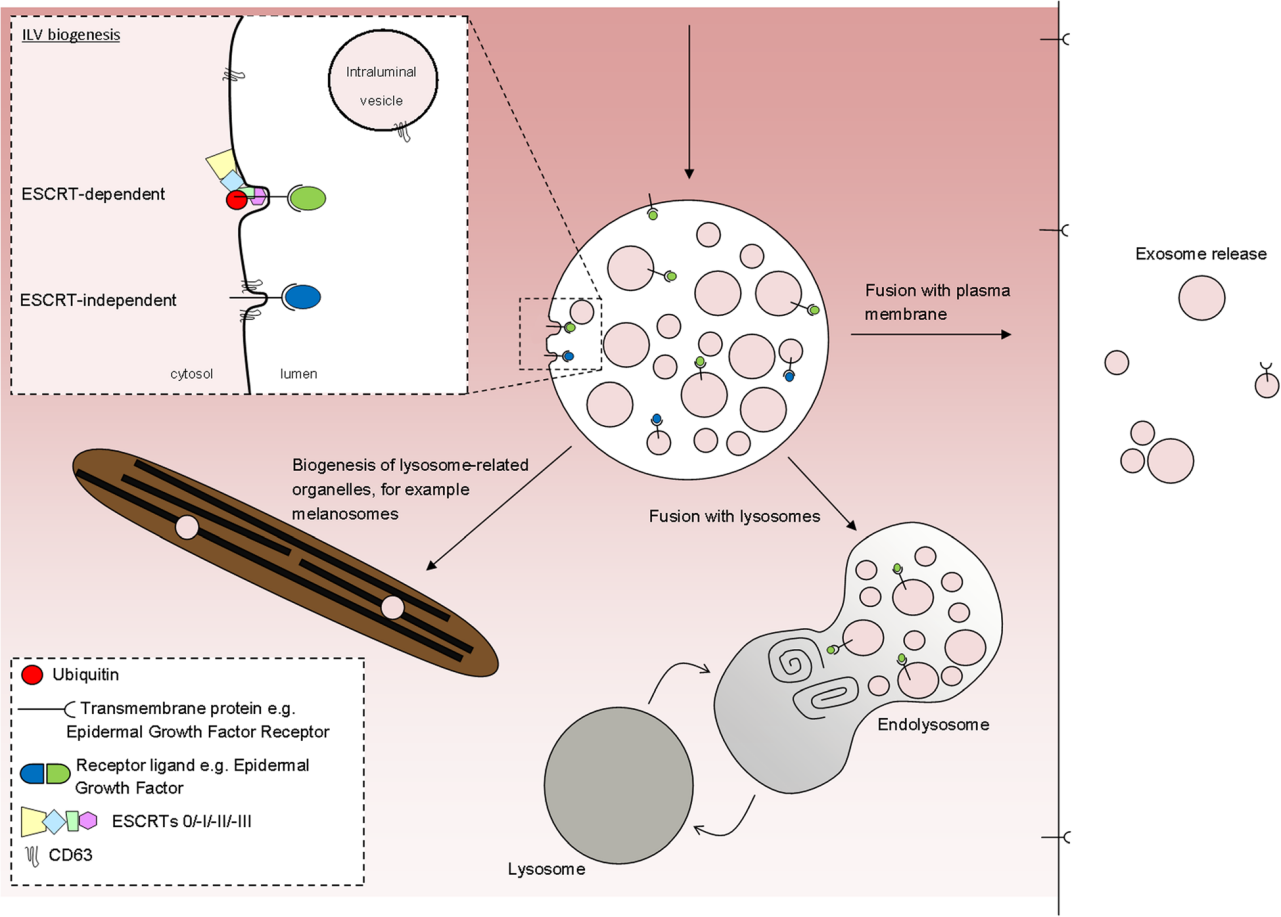
ILVs are generated by invagination of the endosomal membrane and have three possible fates. *Inset*: intraluminal vesicles (*ILV*) are formed by invagination of the endosomal membrane by either ESCRT-dependent or ESCRT-independent mechanisms. Matured endosomes accumulate ILVs within their lumen and have three distinct fates. They may deliver content that contributes to the biogenesis of specialized lysosome-related organelles (for example, melanosomes, Weibel-Palade bodies, azurophilic granules), they may fuse with lysosomes or they may fuse with the plasma membrane where released ILVs are now termed ‘exosomes’

ILVs (and thus exosomes) can be generated at the endosomal limiting membrane by at least two mechanisms, one of which depends on the ESCRT machinery (ESCRT stands for endosomal sorting complexes required for transport) whereas the other is ESCRT-independent (Fig. 2).

The ESCRT machinery consists of a set of cytosolic protein complexes that are recruited to endosomes by membrane proteins that have been tagged, usually with ubiquitin on their cytosolic domains. The ubiquitin tag is recognized by the first of the ESCRT complexes, ESCRT-0, which is thus recruited to the endosomal membrane and passes ubiquitinated cargos to ESCRT-I, one of whose components, Tsg101, also recognizes ubiquitin. The recruitment of the ESCRT machinery acts to both cluster the ubiquitinated cargo proteins on the endosome and induce curvature of the endosomal membrane to form ILVs.

But ILVs are still able to form in the absence of ESCRTs [8], so other means of generating ILVs must exist, although the mechanisms for their generation are less clear. Generation of these ESCRT-independent ILVs requires the tetraspanin CD63—a protein abundant on ILVs but with unclear function [9]—and may be facilitated by cone-shaped bending properties of lipids such as ceramide [10].

## If not all ILVs become exosomes, what determines the fate of an ILV?

The destiny of ILVs is directed by the fate of the MVB they reside in. Confusingly, in addition to different types of ILVs, there are also different types of MVBs [11] and what regulates the fate of these endosomes is another interesting question. MVBs have several potential fates (Fig. 2) and can either fuse with lysosomes (where contents are degraded and recycled), fuse with the plasma membrane (where ILVs are released as exosomes), as I have already mentioned, or contribute to the generation of specialised organelles, such as melanosomes (in melanocytes), Weibel-Palade bodies (endothelial cells), azurophilic granules (in neutrophils) and secretory granules (in mast cells). The levels of cholesterol on MVBs appear to play a part in regulating their fate, cholesterol-rich MVBs being directed to the plasma membrane for exosome release, while cholesterol-poor MVBs are targeted to the lysosome [12].

But what regulates the balance between exosome release and alternative fates of ILVs remains engimatic.

## What about differences between cells: do all cells release exosomes?

Well, not all cells have an endomembrane system, so no. But most mammalian cells contain endomembranes and generate ILVs within MVBs, though remarkably little is known about exosome release in most cell types.

Some cells—for example, the B cells, dendritic cells and mast cells of the immune system—appear to release exosomes constitutively; in fact, most of the data we have on exosomes comes from immune cells. As well as releasing exosomes constitutively, these cells may also be stimulated to secrete exosomes by cellular interactions. For example, murine dendritic cells, which are specialized to activate T lymphocytes, secrete higher levels of exosomes upon interaction with antigen-specific CD4+ T lymphocytes [13]. In fact, lymphocyte interactions generally can be accompanied by exosome release; human T cells (including primary T cells from blood, T cell clones and Jurkat cell lines) release exosomes upon activation of their antigen receptors [14] and B cells release more exosomes upon engagement with antigen-specific CD4+ T cells [15].

Other cell types can be pushed to secrete exosomes by means of calcium ionophores or other stimuli [16, 17], but the extent of physiological exosome secretion in non-immune cells is largely unknown.

## What happens when exosomes reach an acceptor cell?

**Fig. 3:**
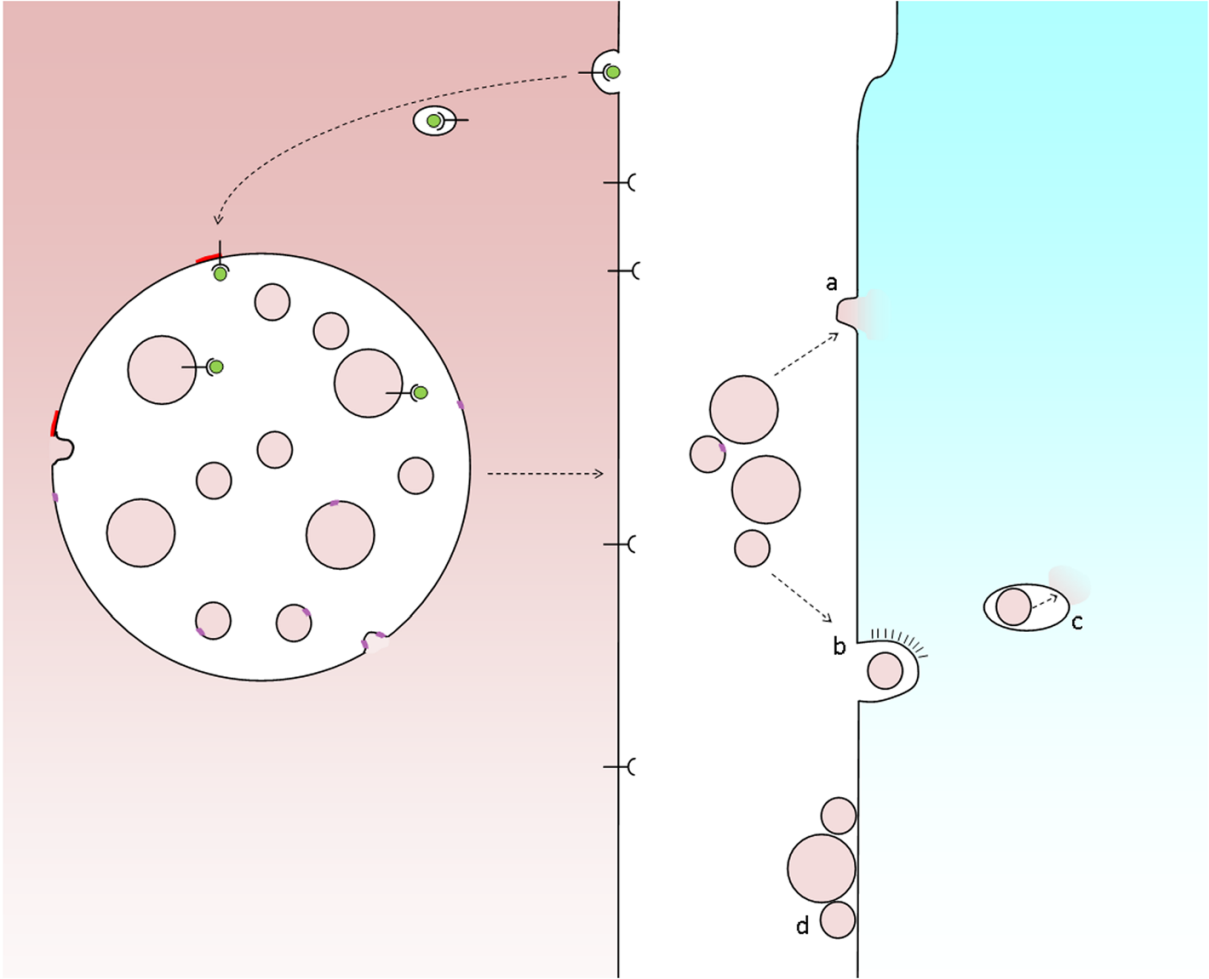
Exosome uptake by recipient cells. Fusion of MVBs with the cell surface releases ILVs as exosomes. In order for exosomes to elicit a response from recipient cells they might either fuse with plasma membrane (**a**) or be taken up whole via endocytosis (**b**), following which the exosome must be delivered to the cytosol, for example, via a back-fusion event (**c**). Alternatively, exosomes may attach to the surface of recipient cells to elicit a signalling response (**d**)

We don’t know exactly. Exosomes that transfer membrane proteins or luminal content to the acceptor cell may be engulfed whole or the exosome membrane may fuse directly with the host plasma membrane (Fig. 3). Alternatively, exosomes may not need to be taken up by cells to elicit a physiological response: follicular dendritic cells, for example, carry on their cell surface exosomes that bear MHC–peptide complexes and other proteins that they do not express and are thereby enabled to activate immune cells with which they interact [18].

For intercellular transmission, various mechanisms of phagocytosis and endocytosis of extracellular vesicles have been described and which mechanism operates may depend upon vesicle size, which may in turn depend upon the cargo carried by the vesicle. In order for material to be released to an acceptor cell, exosomes must fuse with the host cell and this takes place via either direct fusion with the plasma membrane or a ‘back-fusion’ step from within a host endocytic organelle after the exosome has been engulfed. The process of back-fusion is not entirely clear, although it appears to require the unconventional lipid LBPA and protein Alix [19] (and is exploited by anthrax toxin lethal factor to escape from endosomes to the cytosol [20]).

Whether exosomes fuse with target cells or act via interactions with cell-surface proteins, or both, is another fundamental cell biology question that will need to be addressed if we are to understand the functions of exosomes.

## So what are the consequences of all this information transfer? What biological functions have been established for exosomes?

There are many proposed functions for exosomes, the best-established being in immune responses. Exosomes isolated from B lymphocytes and bearing MHC class II molecules were shown in early experiments [4] to activate T lymphocytes in vitro, suggesting that they were communicating with the T lymphocytes in just the way that the parent B cells did. I have already mentioned later work by the same group, who showed that exosomes derived from dendritic cells, which are specialized to activate T cells in the initiation of immune responses, could promote antitumour immune responses in mice [5], exciting interest in the possibility of practical applications.

Or, as with follicular dendritic cells, exosome-associated MHC II can be found on the surface of cell types that neither express MHC II nor secrete exosomes, indicating that exosomes are delivered from one cell type to another [18].

However, exosomes may have roles other than in immune responses as several non-immune cells secrete exosomes. The only physiological role so far reported for non-immune cells is in keratinocyte-derived exosomes, which have been shown to modulate melanin synthesis by increasing the expression and activity of proteins within the melanosomes that modulate skin pigmentation [21].

## How exactly would exosomes from one cell influence the expression and activity of proteins in an acceptor cell?

Exosomes transfer not only protein and lipids but mRNA and microRNA into acceptor cells and these RNAs have been shown in experiments in vitro to have functional effects in recipient cells. For example, exosomes from mice can be transferred to human cells and mRNA can be translated into mouse protein [22]. Similarly, microRNAs—double-stranded RNA fragments that can regulate specific sets of mRNA (and so protein levels)—can act functionally in acceptor cells. The mode of action of exosomes has been a focus of special interest in cancer biology. Exosomes from breast cancer cell lines, for example, have been shown to be enriched for miRNAs relative to nontumorigenic breast cell lines and exposure of normal cells to exosomes derived from breast cancer cell lines increased both cell survival and proliferation, accompanied by loss of expression of some tumour-suppressor proteins [23]. Exosome levels are elevated in the serum of some cancer patients versus controls. However, whether these vesicles are exosomes or other forms of extracellular vesicle, or a mix, is unclear—I have already mentioned this persistent problem in exosome research.

## So exosomes can also contribute to disease?

Yes indeed. As exosomes provide a means of intercellular communication, they may also act as vehicles for ‘bad’ communication or spread. As well as miRNAs in the case of cancer, exosomes have been shown to contain numerous disease-associated cargos—for example, neurodegenerative-associated peptides, such as Aβ [24] (in Alzheimer’s disease), tau [25] (in numerous neurodegenerative diseases), prions [26] (in transmissible spongiform encephalopathies), alpha-synuclein [27] (in synucleinopathies, including Parkinson’s disease) and superoxide dismutase 1 [28] (in amyotrophic lateral sclerosis). Exosomes have thus been suggested to be propagators of neurodegenerative protein spread, although some cargos are easier to envisage than others.

Of the neurodegenerative-associated proteins, only some are integral membrane proteins, that is, proteins inserted into lipid bilayers, rather than cytosolic. Sorting of proteins into ILVs (and thus exosomes) is easier to envisage for membrane proteins, where tags such as ubiquitin regulate where they end up. So far, the presence of both Aβ [29] and PrPc [26] has in fact been shown in ILVs, though this has not been demonstrated for other membrane proteins, such as alpha-synuclein and tau.

The mechanism whereby cytosolic proteins may be sorted to ILVs/exosomes, however, is not clear. In order for cytosolic proteins to become concentrated in ILVs, they would require positive incorporation and sorting, possibly by membrane-associated components on endosomes. All we can say is that there is evidence that this does in fact happen; cytosolic factors such as miRNAs are enriched in exosomes relative to cytosol, indicating that sorting must occur whereby certain miRNAs are concentrated and others are not [30].

The means by which disease-associated factors spread between cells remains poorly understood and exosomes would provide a means for such transmission. The presence of exosomal proteins, such as Alix, in association with Alzheimer’s senile plaques strengthens the circumstantial case for exosomes as a mediator in such spread. The hope is that having a means to regulate exosome release and spread may be useful in combatting some of these diseases but much more basic biology needs to be established before then.

## Now I’m confused—what determines what exosomes contain?

Exosomes will contain whatever is sorted into them during their formation (as ILVs). For membrane proteins, this usually occurs through ubiquitination, which acts as a substrate for recruitment of the ESCRT machinery and subsequent generation of ESCRT-dependent ILVs.

The mechanisms that concentrate cytosolic factors are currently unknown. Although it seems clear that miRNAs, for example, are enriched relative to the amount in their parent cells, and are not randomly incorporated into exosomes, it is not clear how some are enriched more than others. There are currently a few hypotheses for miRNA sorting, including sorting via sumoylated heterogeneous nuclear ribonucleoproteins [31] or by a miRNA-induced silencing complex (miRISC) [32].

Because of the difficulties in separating exosomes from other extracellular vesicles, it is likely that some cargos reported to be enriched in ‘exosomes’ may in fact be contained in contaminant vesicles that are not exosomes. While many researchers are very stringent about applying the labels ‘exosomes’ and ‘extracellular vesicles’ correctly, others unfortunately are not. In addition, as I have said before, cytosolic proteins are likely to be found in exosome preparations because the exosome lumen is made of cytosol.

## So how exactly can you be sure that a given extracellular vesicle is an exosome and not something else?

This is an interesting question that has a complex answer. Ideally, an intracellular compartment is identified by a specific biological marker, as, for example, in the case of the Golgi, nucleus or mitochondria, all of which carry proteins not found, or found at much lower levels, elsewhere.

One problem is that ILVs, and thus exosomes, represent an intermediate compartment of an intermediate. MVBs are not static organelles but rather undergo continuous maturation, in the course of which they gain and lose proteins. There will never be an exclusive marker for exosomes because any cargo on the ILV/exosome membrane must first be on the limiting membrane of the endosome and anything found inside must first come from the cytosol. A cargo may be concentrated on ILVs/exosomes but it will also be elsewhere. CD63 could be thought of as a pseudo-marker for exosomes. ILVs and exosomes are enriched in several such tetraspanins and my colleagues and I have show that CD63 is required for ESCRT-independent ILV formation [9]. Alix also appears to be concentrated in ILVs/exosomes [33], as does Tsg101, a component of ESCRT-I, which has been used as a marker of exosomes in numerous studies [33, 34], although the presence of Tsg101 in ILVs or exosomes does not fit with conventional models of ILV formation. Although Tsg101 is involved in ESCRT-dependent ILV formation, as mentioned earlier, it, along with other ESCRT components, should disassociate from the endosomal membrane prior to an ILV pinching off the endosomal membrane to allow it to participate in further events [35]. Exactly when ESCRT-I components ‘fall off’ the membrane is unknown but it is conventionally thought to be prior to ILV formation, so Tsg101 should remain cytosolic and available for subsequent rounds of ILV formation. It is possible that some Tsg101 may be ‘swallowed’ into the forming ILV lumen, but levels should be negligible.

## So are you saying there is no reliable marker for endosomes?

There may not be—not a single reliable one. Ultimately, perhaps the best method of defining exosomes biochemically may be through a combination of markers, including tetraspanins, Alix and others, with a concomitant exclusion of resident plasma membrane proteins. Although ILVs/exosomes will by their nature contain some plasma membrane proteins and the plasma membrane will contain some ILV/exosomal proteins, it should be possible to define relative levels and/or enrichment of proteins of exosomes that distinguish them from other microvesicles. Cargos such as MHC II from B cells and other cell type-specific antigens may also help to distinguish exosomes from other forms of extracellular vesicle. Common exosomal cargos include tetraspanins (CD63, CD81, CD9), antigen presentation molecules (MHC I and MHC II) and others (Alix, flotillin-1). An online database exists [36] where proteins, lipids and RNA are catalogued from published and unpublished exosomal studies.

## If they are so hard to characterize reliably, how are exosomes isolated and studied?

Exosomes are rarely imaged by conventional methods as they are too small to be resolved by fluorescence microscopy and their release may be a rare event. A few studies have imaged exosome release occurring in cell cultures by various electron microscopic techniques but, more commonly, exosomes are pooled from cellular supernatant or animal fluids. Traditionally, they have been isolated by differential centrifugation from culture medium whereby larger contaminants are first excluded by pelleting out through increasing speeds of centrifugation before exosomes, small extracellular vesicles and even protein aggregates are pelleted at very high speeds (~100,000 × *g*) [37]. These preparations therefore represent an enrichment rather than a purification. Enriched preparations are commonly analysed by biochemistry, mass spectrometry or electron microscopy. Electron microscopy of isolated fractions as ‘whole mounts’ make it possible to immuno-label vesicles, with the limitation that isolated preparations do not provide the same internal controls as labelling sections of cells. Remarkably little attention has been paid to the characterization of exosomes, although efforts are being made to repair this omission with guidelines and criteria for defining groups of extracellular vesicles [38].

## What would you say are the most important issues in exosome research?

Without doubt the single most important issue is actually understanding the biological significance of these structures. With so little known about their basic physiological functions, it may seem hard to understand how exosomes have been implicated in the pathogenesis of so many disparate disease states. Fundamental questions remain about exosome generation, fate and normal function but, ultimately, in order to understand exosomes, one must first understand ILVs, a fact that is too often overlooked. Meanwhile, it is important that publications on exosomes give a careful and explicit account of the criteria used for distinguishing them from other extracellular vesicles to avoid confusing the field and encouraging scepticism.
